# Low BMD affects initial stability and delays stem osseointegration in cementless total hip arthroplasty in women: A 2-year RSA study of 39 patients

**DOI:** 10.3109/17453674.2012.678798

**Published:** 2012-04-24

**Authors:** Hannu T Aro, Jessica J Alm, Niko Moritz, Tatu J Mäkinen, Petteri Lankinen

**Affiliations:** Orthopaedic Research Unit, Department of Orthopaedic Surgery and Traumatology, Turku University Hospital and University of Turku, Turku, Finland

## Abstract

**Background and purpose:**

Immediate implant stability is a key factor for success in cementless total hip arthroplasty (THA). Low bone mineral density (BMD) and age-related geometric changes of the proximal femur may jeopardize initial stability and osseointegration. We compared migration of hydroxyapatite-coated femoral stems in women with or without low systemic BMD.

**Patients and methods:**

61 female patients with hip osteoarthritis were treated with cementless THA with anatomically designed hydroxyapatite-coated femoral stems and ceramic-ceramic bearing surfaces (ABG-II). Of the 39 eligible patients between the ages of 41 and 78 years, 12 had normal systemic BMD and 27 had osteopenia or osteoporosis. According to the Dorr classification, 21 had type A bone and 18 had type B. Translational and rotational migration of the stems was evaluated with radiostereometric analysis (RSA) up to 2 years after surgery.

**Results:**

Patients with low systemic BMD showed higher subsidence of the femoral stem during the first 3 months after surgery than did those with normal BMD (difference = 0.6, 95% CI: 0.1–1.1; p = 0.03). Low systemic BMD (odds ratio (OR) = 0.1, CI: 0.006–1.0; p = 0.02), low local hip BMD (OR = 0.3, CI: 0.1–0.7; p = 0.005) and ageing (OR = 1.1, CI: 1.0–1.2; p = 0.02) were risk factors for delayed translational stability. Ageing and low canal flare index were risk factors for delayed rotational stabilization (OR = 3, CI: 1.1–9; p = 0.04 and OR = 1.1, CI: 1.0–1.2; p = 0.02, respectively). Harris hip score and WOMAC score were similar in patients with normal systemic BMD and low systemic BMD.

**Interpretation:**

Low BMD, changes in intraosseous dimensions of the proximal femur, and ageing adversely affected initial stability and delayed osseointegration of cementless stems in women.

Cementless techniques in total hip arthroplasty (THA) were originally designed for patients with normal bone structure and normal healing capacity. Osseointegration of uncemented components is based on new bone ingrowth and ongrowth, which is inhibited by excessive micromotion (Pilliar et al. 1986). Thus, high initial stability is one of the key factors for rapid osseointegration of an implant. Along with biomechanically optimized implant designs and bioactive coatings for enhanced bone ingrowth, the indications have gradually been expanded to include even THAs in elderly patients with impaired bone quality and limited healing capacity (Keisu et al. 2001, Kelly et al. 2007, Meding et al. 2010, Mäkelä et al. 2010, Thillemann et al. 2010).

Osteoporosis is common in postmenopausal women with osteoarthritis (OA) of the hip (Glowacki et al. 2003). Cementless THA techniques have never been systematically screened for appropriate indications in osteoporotic patients, although poor bone quality may jeopardize the initial stability of cementless stems. The bone-implant interface must withstand high shear stresses of physiological loading, and poor quality of periimplant bone may also jeopardize the long-term success of osseointegration (Gabet et al. 2010).

We have used radiostereometric analysis (RSA) for evaluation of the success of cementless THAs in a population of female patients with primary OA of the hip. The study hypothesis was that the bone quality of postmenopausal women dictates the early stability of anatomically designed femoral stems.

## Methods

### Study design

This 2-year single-center RSA study involved female patients who underwent cementless THA for primary hip OA. The patient population was screened preoperatively for the presence of undiagnosed primary and secondary osteoporosis and vitamin D deficiency (Mäkinen et al. 2007). All patients received an RSA-marked hydroxyapatite-coated hip implant (Anatomic Benoist Girard II (ABG-II); Stryker). Ceramic heads and ceramic liners made of aluminium oxide (Al_2_O_3_) were used in order to avoid any confounding effects of wear particles on implant osseointegration.

Preoperative systemic BMD measured by dual-energy X-ray absorptiometry (DXA) was designated the principal indicator of baseline systemic skeletal status. 10 anatomical areas were measured at the lumbar spine, proximal femurs, and the distal non-dominant forearm. Patients with a T-score of less than –2.5 at any of the 10 anatomical sites were classified as being osteoporotic and patients with T-scores of between –1 and –2.5 were classified as being osteopenic. In the data analysis, osteoporotic and osteopenic patients were combined in one group designated “low BMD”. Patients with T-scores higher than –1 in all 10 anatomical areas were designated “normal BMD”. The primary outcome of the study was the effect of systemic BMD on RSA-measured stem migration, as evaluated by comparing the low BMD group vs. the normal BMD group.

The study protocol was approved by the Ethics Committee of the Hospital District of Southwest Finland (# 4/2002 §76). Informed consent was obtained from all patients.

### Inclusion and exclusion criteria

The inclusion criteria were (1) a generally healthy woman aged less than 80 with advanced primary hip OA, and (2) and unremarkable medical history. Exclusion criteria included rheumatoid arthritis or any other inflammatory arthritis, hereditary skeletal disease, untreated parathyroid disease, ongoing osteoporosis or corticosteroid therapy or any other medication affecting bone metabolism, and severe undiagnosed primary osteoporosis (T-score less than –3.5).

**Figure 1.**
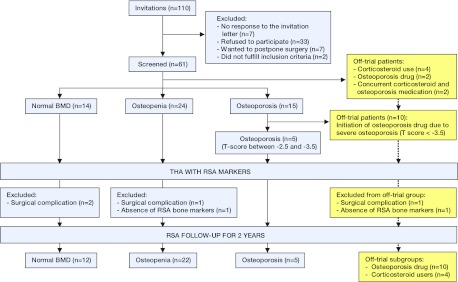
Study design.

Potential participants were identified from patients scheduled to have cementless THA at Turku University Hospital. From the 110 invitations sent out by mail, 70 positive responses were received and eventually 61 subjects were screened for the study (Figure 1). The study population can be considered to represent a normal female population with advanced OA of the hip in southwest Finland.

Of the 43 patients who fulfilled the study criteria, 3 were excluded due to a surgical complication (periprosthetic fracture) and 1 due to the absence of bone RSA markers. Thus, 39 patients were enrolled (Table 1). All of them completed the 2-year follow-up.

**Table 1.** Demographic data

No. of cases	39
Age, years	64 (41–78)
BMI, kg/m**^2^**	31 (SD 6)
Previous fractures	8
Systemic BMD	
normal bone	12
osteopenia	22
osteoporosis	5
Dorr classification	
type A	21
type B	18
type C	0

During the screening process, it became evident that the study would benefit from a parallel off-trial follow-up of excluded patients with severe undiagnosed primary osteoporosis requiring the initiation of bisphosphonate therapy, osteoporotic patients with ongoing bisphosphonate therapy, and patients who had corticosteroid treatment at baseline. Following an accepted amendment by the Ethics Committee, the off-trial patients followed the same RSA and clinical protocol as the study patients (Figure 1). 1 of the off-trial subjects was excluded because of a surgical complication (periprosthetic fracture) and another was excluded due to the absence of RSA bone markers. 2 off-trial patients with concurrent use of a bisphosphonate and corticosteroids were not included in the data analysis.

### RSA-marked stem and surgery

**Figure 2.**
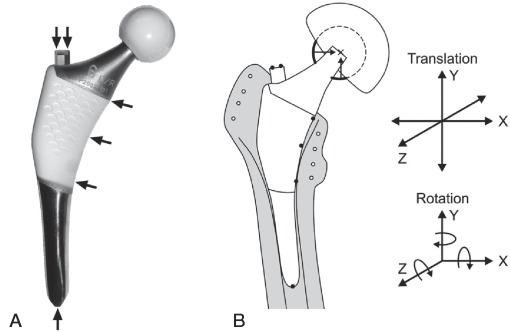
A. The ABG-II stem with proximal HA coating and 3 medial tantalum RSA markers (arrows). There were 2 additional RSA markers on the shoulder of the stem and 1 marker in the distal tip. B. Schematic drawing of the prosthesis with tantalum RSA markers in the prosthesis and in the surrounding bone, with the coordinate system for RSA analysis of 3D micromotion of the implant.

The anatomically shaped ABG-II stem (Figure 2) is intended to follow the anatomical intraosseous contours of the proximal femur. Based on finite element modeling, the stem ensures that the load transfer pattern imitates (as closely as possible) the natural distribution of bone stress within physiological limits (van Reitenbergen and Huiskes 2001). Indeed, the stem appears to cause only a temporary reduction of total periprosthetic BMD even in the presence of local bone loss in Gruen zone 7 (Alm et al. 2009). For the current study, the stem was marked by the manufacturer with 4 1.0-mm tantalum markers (1 at the tip and 3 on the side of the body) for RSA analysis (Figure 2). In addition, a peg containing 2 tantalum markers was inserted into the shoulder of the stem during surgical implantation. During surgery, 5–8 tantalum markers (0.8 mm) were inserted into the greater and lesser trochanters using the UmRSA Injector (RSA Biomedical, Umeå, Sweden).

### RSA

After surgery, the patients were instructed to do partial weight bearing for 6 weeks, followed by full weight bearing. RSA examination using uniplanar technique (Kärrholm et al. 1997) was performed within 7 days of surgery (baseline) and repeated at 3, 6, 12, and 24 months. The setup for RSA imaging was standardized thorough studies of THA phantom models (Mäkinen et al. 2004). Image analyses were performed according to the RSA guidelines (Valstar et al. 2005).

At each time point, migration of the stem in terms of 3D rotation and translation were determined in relation to the postoperative baseline examination using UmRSA v. 6.0.3.7 software (RSA BioMedical Innovations AB, Umeå, Sweden). The linear movements of the stem were analyzed as translations along 3 axes (x, y, z) and the angular movement was analyzed as rotations around 3 axes (x, y, z) (Figure 2). In addition, total translation and rotation vectors were calculated as described previously (Kaptein et al. 2007).

In addition to the fixed tantalum markers in the stem, the center of the femoral head was used as one marker. Stability and adequate distribution of the markers were assessed by calculating mean error of rigid body fitting (ME) and condition number (CN) (Valstar et al. 2005). The upper limit of the mean error of rigid body fitting was kept at around 0.35 and the upper limit of condition number was kept at 150. All patients were subjected to duplicate examinations at some of the follow-up time points, with the patients repositioned between examinations. The precision was calculated as 1.96 standard deviations of the mean value of differences between duplicate examinations. Based on normal distribution of each type of movement, this represents 95% of the total error distribution (Kärrholm et al. 1997, Bragdon et al. 2002). Based on all duplicate examinations and the use of stem markers at 3 locations (tip, shoulder, and medial border), the detection limit of the selected RSA parameters was 0.42 mm for y-translation, 0.40 mm for the translation vector, 1.81 degrees for y-rotation, and 1.32 degrees for the rotation vector. Accordingly, micromotions of the stems exceeding these values were classified as migration.

When the precision was calculated from those duplicate examinations in which the RSA markers on the medial side of the stem were not visible and the femoral head was used as the additional marker, the precision values were better (0.17 mm for y-translation and 1.20 degrees for y-rotation), corresponding to those reported by other users of the UmRSA system. Based on a clinical point of view, the selected main parameters were translational migration along the y-axis (y-translation; stem subsidence) and rotational migration along the y-axis (y-rotation; anteversion-retroversion), and the translation vector and the rotation vector were used as indicators of overall 3D micromotion.

### Stem osseointegration

Based on the presence of dichotomy on migration patterns of cemented THA cups (Aspenberg et al. 2008), it might also be useful in RSA studies of cementless THA to compare the number of patients above a certain cutoff level rather than simply to compare group means. Thus, we used the RSA data also for assessment of implant osseointegration at the level of the individual patient. The detection of cessation of translational and rotatory migration was used to define the exact time point at which a patient was found to have a stable (osseointegrated) stem. At each time point, stems were classified as fixed (osseointegrated) or migrating by comparing the stem position measured to the stem position at the preceding time point. If the micromotion measured exceeded the detection limit, the stem was defined as migrating. Normal osseointegration was defined as stabilization of a stem by 3 months. Delayed osseointegration was defined as migration of a stem for up to 6 months. Correspondingly, stems that migrated for up to 12 months were considered to show late osseointegration. The stems that showed continuous migration for 12–24 months were classified as being unstable.

### Geometry of the proximal femur

The geometry of proximal femurs was evaluated by qualitative classification into three distinct pattern types (Dorr type A, B, and C) (Dorr et al. 1993) and by calculation of the canal flare index (CFI). CFI was calculated from anteroposterior digital radiographs of the hips (Noble et al. 1995). The metaphyseal width 20 mm proximal to the most prominent point of the lesser trochanter (D) and the width of the intramedullary femoral isthmus (G) were measured using a computerized method. CFI was calculated as the ratio of D to G. Using the classification system, the canal shapes of the femurs were normal (CFI 3–4.7) in 3 subjects, stovepipe in 29 subjects (CFI < 3), and champagne-flute in 3 subjects (CFI > 4.7).

### Assessment of clinical outcomes

Harris hip score was used in physician assessment of localized pain and physical functioning. WOMAC questionnaire was used as a validated disease-specific tool for patient-related outcome.

### Statistics

The magnitude of stem migration is presented as mean (SD). Stem migration from baseline to 3 months was analyzed with paired t-test, while the overall migration during the 24-month study period was analyzed using repeated measures ANOVA. To investigate whether the time-related stem migration was different in the 2 study groups, “group” was used as cofactor in the repeated measures analysis. Differences between the 2 groups were further investigated at each time point using Student’s t-test and results are presented as the mean difference with 95% confidence interval (CI) for the difference, along with p-values.

Differences between the 2 study groups in the time point of implant osseointegration (stable/unstable stem at 3, 6, 12, or 24 months) were analyzed using Kaplan-Meier time-to-event estimates. The only censored observations were the unstable stems at 24 months. The effect of demographic parameters on osseointegration of the implant was analyzed with a logistic-regression model adjusted for age. The parameters investigated were age, BMI, local BMD and T-score of the operated hip, and canal flare index. Odds ratios (ORs) with CI and p-values were used to estimate the effect on stem stabilization. The data did not include censored observations apart from patients whose stems had not reached osseointegration after 24 months.

Time-related changes in WOMAC and Harris hip scores were evaluated with repeated measures ANOVA. Differences in scores between the 2 study groups at each time point were investigated with Student’s t-test and are presented as mean differences with CI and p-values.

The off-trial patients were analyzed as 2 groups (1 group of patients with bisphosphonate treatment and 1 group using corticosteroids). Time-related stem migration in the off-trial patients was compared with that in the study patients using repeated measures ANOVA with “group” as cofactor. The magnitude of stem migration at 3 and 24 months in the 2 off-trial groups was compared to those in the 2 main study groups using Mann-Whitney U test, since the data for the off-trial groups did not fulfill the criteria of normal distribution and equal variance.

In all analyses with RSA parameters as continuous variables, RSA data values lying outside 1.5 times the interquartile range from the first and the third quartiles were identified as outliers, and they were excluded from the analysis. The number of outliers varied between 2 and 4 depending on the RSA parameter and the time point. 1 patient who showed an abrupt 8.5 degrees of rotation of the stem by 3 months followed by minimal further migration up to 2 years was treated as an outlier and was excluded from the statistical analysis. For all repeated measurements, normal distribution of the data was confirmed with the Kolmogorov-Smirnov test and sphericity was checked with Mauchly’s test. In cases in which sphericity was not fulfilled, the Huynh-Feldt correction was used. A significance level of 0.05 was used.

## Results

### Average stem migration

The migration of the stems was calculated as an average for all patients (normal and low BMD groups combined) in order to get a reference value for comparison to published RSA studies. The principal stem migration, both in translation and rotation along the y-axis, took place within the first 3 months after surgery, but not thereafter. At 3 months, the mean stem subsidence was 0.9 (0.8) mm (compared to baseline, p < 0.001) and the mean rotation was 0.8 (2.0) degrees (compared to baseline, p = 0.03). Stem subsidence exceeding the detection limit (0.42 mm) was found in 27 of the 39 patients. Rotation was detected in 21 patients, retroversion in 17, and anteversion in 4.

### Effect of systemic BMD on stem migration

Preoperative systemic BMD was predictive of the magnitude of stem subsidence (p = 0.007). This statistically significant difference between the groups was evident at 3 months and remained unchanged during the 24-month follow-up period (Table 2). The group of patients with normal BMD showed a narrower standard deviation than those with low BMD (Table 2).

**Table 2.** Comparison of time-related changes in RSA migration and functional scores in THA patients with normal and low BMD

	Normal BMD	Low BMD			
	(n = 12)	(n = 27)			
Time point (months)	Mean (SD)	Mean (SD)	Difference	95% CI	p-value **^d^**
Stem subsidence **^a^**					
3	–0.5 (0.5)	–1.1 (0.9)	0.6	0.1 to 1.2	0.02
6	–0.5 (0.6)	–1.3 (1.1)	0.8	0.1 to 1.5	0.02
12	–0.5 (0.6)	–1.2 (1.1)	0.8	0.06 to 1.4	0.03
24	–0.5 (0.6)	–1.3 (1.0)	0.8	0.1 to 1.5	0.02
Stem rotation **^b^**					
3	1.2 (0.8)	1.6 (1.7)	–0.5	–1.5 to 0.6	0.4
6	2.0 (1.1)	1.6 (1.7)	0.4	–0.7 to 1.6	0.5
12	2.0 (1.4)	1.7 (1.8)	0.3	–1.0 to 1.5	0.6
24	1.8 (1.4)	1.8 (2.0)	0.03	–1.3 to 1.3	0.6
Harris hip score **^c ^**					
Preop.	49 (13)	51 (17)	–1.3	–13 to 11	0.8
3	75 (22)	75 (17)	0.02	–14 to 14	1.0
6	87 (13)	85 (13)	2.1	–7 to 12	0.7
12	87 (10)	88 (12)	–0.7	–9 to 7	0.9
24	86 (17)	85 (13)	1.1	–9 to 12	0.9
WOMAC score **^c ^**					
Preop.	47 (13)	53 (17)	–6.2	–21 to 9	0.4
3	26 (21)	25 (13)	1.1	–11 to 13	0.9
6	19 (11)	20 (13)	–1.5	–11 to 8	0.8
12	10 (5)	16 (11)	–5.5	–13 to 2	0.1
24	13 (9)	17 (16)	–4.2	–15 to 6	0.4

**^a ^**y-translation

**^b ^**y-rotation

**^c ^**Global p < 0.001 for the time-related change (repeated measures ANOVA).

**^d^** Student’s t-test.

**Figure 3.**
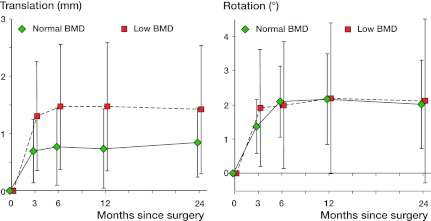
Stem migration determined by RSA in women with normal or low systemic BMD. A. Total translational migration expressed as translation vector. B. Total rotational migration expressed as rotation vector. The values represent mean (SD).

For the translation vector, representing total translational migration in different axes, the difference between the 2 groups was not statistically significant (p = 0.1) (Figure 3A). There was a time-related change in the translation vector at 3 months (p < 0.001 for both groups), with additional moderate migration between 3 and 24 months (p = 0.03 for both groups).

Both groups showed statistically significant rotation along the y-axis at 3 months (p < 0.001), but not thereafter. Preoperative systemic BMD did not affect the magnitude of y-rotation (p = 0.9) (Table 2).

From baseline to 3 months, there was a statistically significant change in rotation vector, representing total rotational migration along different axes, in both groups (p < 0.001 for both) (Figure 3B). Patients with normal BMD showed a moderate time-related increase in rotation vector between 3 and 24 months (p = 0.03). In the patients with low BMD, the rotation vector stabilized by 3 months with no further time-related changes. Even so, the rotation vector was similar between the 2 groups (p = 0.8) (Figure 3).

### Osseointegration of the stem

RSA assessment of stem osseointegration at the individual level was mainly performed on data from the total translation and rotation vectors. Translational stability (total translational migration ≤ 0.40 mm) could be detected in 7 patients at 3 months, representing the time course of normal osseointegration. In the majority of patients (n = 24), the stem had stabilized by 6 months, corresponding to delayed osseointegration. 4 patients showed late osseointegration at 12 months. 4 patients showed detectable translation of the stem at 24 months, indicating instability.

Stem stability evaluated with the total rotational migration (rotation vector) showed that 17 patients achieved osseointegration at 3 months. In the remaining 22 patients, rotation exceeded the detection limit (1.32 degrees) at 3 months. 3 patients showed delayed osseointegration (cessation of rotation at 6 months) and as many as 8 patients had late osseointegration (cessation of rotation at 12 months). At 24 months, rotational migration was still detectable in 2 patients.

Combining all 4 RSA parameters (translation vector, rotation vector, y-translation, and y-rotation), detectable stem migration (translation or rotation, or both) was found in 7 patients of 39—as a sign of unstable stems at 24 months. 2 of them had normal BMD and 5 had low BMD. All 7 patients reported slight or moderate pain in the operated hip. None of them had classical signs of mechanical stem loosening on radiographs at 24 months.

The observed differences in time points for osseointegration between patients with normal BMD and those with low BMD were analyzed using Kaplan-Meier time-to-event estimates. A statistically significant difference in total translational migration (translation vector) was found between the 2 groups (p = 0.02). The patients with low BMD had a 4% probability of osseointegration at 3 months, as compared to a 50% probability in patients with normal BMD. Evaluation of rotational stability (rotation vector) showed no differences in estimates between the 2 groups. The probability of osseointegration within 3 months was 42% in the normal BMD group and 44% in the low BMD group.

### Logistic regression risk analysis for delayed osseointegration

Logistic regression analysis was performed to evaluate the effect of demographic parameters on the time point of stem osseointegration. Low systemic BMD (OR = 0.1, CI: 0.006–1.0; p = 0.02), low local hip BMD adjusted for age (OR = 0.3, CI: 0.1–0.7; p = 0.005) and ageing (OR = 1.1, CI: 1.0–1.2; p = 0.02) were found to be risk factors for delayed translational osseointegration. Ageing and low canal flare index were found to be risk factors for delayed rotational osseointegration (OR = 3, CI: 1.1–9; p = 0.04 and OR = 1.1, CI: 1.0–1.2; p = 0.02, respectively).

### Subjective evaluation of functional outcome

Both Harris hip score and WOMAC score improved during the 2 years of follow-up (p < 0.001 for both) (Table 2). Preoperative systemic BMD did not affect the improvement in scores (p = 0.9 for Harris hip score and p = 0.8 for WOMAC score).

### Stem migration in the off-trial subgroups

In the off-trial subgroups of osteoporotic patients under bisphosphonate therapy and patients with corticosteroid treatment, stem migration followed the same patterns as in the study population. In the statistical analysis, there was no significant difference in average migration compared to the study groups. In the corticosteroid users, stems showed osseointegration within 6 months, suggesting that there was no apparent influence of corticosteroids on the stabilization of cementless stems. In the bisphosphonate subgroup, stems showed osseointegration within 6 months in 7 of 10 patients. Only 1 of them had an unstable stem at 24 months.

## Discussion

Osteoporosis may have 4 major potential complications in cementless THA: increased migration and subsequent loss of the optimal position of the stem, delayed osseointegration of the stem due to increased migration, an increased risk of periprosthetic fracture, and a risk of late loosening due to mechanical failure of ingrown trabecular bone. Our study shows that low systemic BMD, geometric changes in the proximal femur, and ageing may indeed increase initial migration and delay osseointegration of cementless femoral stems.

Previous RSA studies have shown that excellent primary stability of uncemented femoral stems with anatomical, non-anatomic straight, or custom designs can easily be achieved in patients with good bone quality (Søballe et al. 1993, Kärrholm et al. 1994, 2002, Bottner et al. 2005, Grant et al. 2005, Thien et al. 2007). These studies have involved selected patient populations with both sexes, usually less than 65 years of age. The average amount of stem subsidence has been reported to be minimal (ranging from zero to 1.00 mm). We used a different approach. We mainly concentrated on postmenopausal women with a high incidence of undiagnosed primary and secondary osteoporosis (Mäkinen et al. 2007). We found that these patients are at risk of increased migration of their uncemented stems. The subsidence measured in patients with low BMD (mean 1.0–1.3 mm) does not even represent the true worst-case scenario because the patients with undiagnosed severe osteoporosis were excluded. The average subsidence in the current series (0.9 mm) corresponds to that reported by Ström et al. (2007) and by Campbell et al. (2011) in their studies of straight stem designs. In their series of 27 patients (18 women) with a median age of 70 years, Campbell et al. recognized a striking variability in the degree of subsidence within the first 6 months, which they assumed to be related to differences in the quality of the initial stem fixation or in the quality of bone.

Anatomical ABG stems, which are designed to accommodate the natural geometry of the proximal femur (van der Wal et al. 2008), would be expected to be highly stable in patients with normal anatomy of the proximal femur. Indeed, the anatomic ABG-I stem has shown minimal subsidence at 1 year (mean 0.17 mm) in a study population of 10 men and 9 women with a mean age of 54 years who were subjected to partial weight bearing after hip replacement (Thien et al. 2007). To our knowledge, there have been no previous data on RSA migration of the ABG-II stem, which is a modified successor of ABG-I. However, in accordance with the previous studies on the ABG-I, our results with the ABG-II stem showed a median degree of subsidence of 0.17 mm (at 2 years) in patients with normal BMD.

Our precision values were higher than previously reported by other laboratories with the same UMRSA system. We have paid attention to standardization of the set-up for RSA imaging (Mäkinen et al. 2004). For RSA measurements, the short ABG-II stem turned out to be demanding. However, the precision values (0.17 mm for y-translation and 1.20 degrees for y-rotation) were closer to those from previous studies, when the femoral head was used as the additional marker.

The geometric changes in the proximal femur, classified by Dorr et al. (1993), occur in ageing women (60–90 years) but not in men (Noble et al. 1995). We have found type-C morphology only in female THA patients with osteoporosis, while patients with osteopenic levels of BMD mainly have type-B morphology (Mäkinen et al. 2007). In the current study, including patients with only type-A or type-B morphology, the measurement of Noble’s canal flare index served as a predictor of delayed rotational stem stability. This observation suggests that even minor changes in the intraosseous dimensions of the proximal femur can indeed adversely affect the initial stability of anatomically designed femoral stems. Our result agrees with the conclusion of Noble et al. (1995) that cementless femoral stems of one standard shape cannot provide a close fit to the endosteal contours of elderly women.

Our results may not be applicable to femoral stems of straight non-anatomic designs relying on 3-point fixation. There have been attempts to shape straight tapered stems for better canal fit in patients with different femoral morphologies (Omlor et al. 2010) and even to custom-design femoral stems (Muirhead-Allwood et al. 2010), but standard straight double-wedge stems may work in most patients with type-C morphology (Kelly et al. 2007, Meding et al. 2010).

Periprosthetic fractures have emerged as a leading postoperative complication in cementless THA (Hailer et al. 2010). The tapered shape of press-fit stems, female sex, and increased age—the latter two probably confounded by osteoporosis—have been suggested to be independent risk factors for fractures (Lindahl 2007, Davidson et al. 2008). The ABG-II stem in particular has shown an increased risk of early periprosthetic fractures (Mäkelä et al. 2010). In the current series, intraoperative or early postoperative periprosthetic fractures (in 4 of 61 patients) were distributed evenly in patient groups with different BMDs. In fact, periprosthetic fractures may occur independently of the patient’s BMD and the issue may be related to implant-specific learning curves (Hailer et al. 2010).

In the proximal femur, osteoporosis and ageing are known to cause (1) reduced density of the trochanteric cancellous bone (Moritz et al. 2011), (2) increased intracortical porosity and endosteal trabeculation (Zebaze et al. 2010), and (3) geometric changes in the femoral canal (Noble et al. 1995, Hartman et al. 2009). Our recent analysis (Moritz et al. 2011) showed that the impaired quality of local intertrochanteric cancellous bone of the proximal femur is not associated with increased stem migration, suggesting that the primary effect of osteoporosis on stem migration is related to endosteal/intracortical changes in the proximal femur and is partly due to the changes in the intraosseous dimensions of the proximal femur. This would also agree with recent CT studies (Mueller et al. 2010) that have shown that common press-fit, tapered-design femoral components rely for stability on cortical contact and not on pure metaphyseal load transfer of the intertrochanteric cancellous bone region, as hypothesized by the original designers of the stems (van Rietbergen and Huiskes 2001).

The next step will be determination of the long-term performance of cementless stems in osteoporotic female patients. Without doubt, repeated RSA studies and functional evaluation after 5 and 10 years will reveal the real clinical impact of the increased migration and delayed osseointegration found in the current study.
